# A novel momentum-based intervention sustains real-life participation in a social movement

**DOI:** 10.1038/s41598-026-43735-x

**Published:** 2026-03-17

**Authors:** Noa Cohen-Eick, Eric Shuman, Yossi Hasson, Martijn van Zomeren, Eran Halperin

**Affiliations:** 1 Department of Psychology, Hebrew University, Jerusalem, Israel; 2 Department of Psychology, The University of Virginia, Charlottesville, VA USA; 3 aChord Center, The Hebrew University of Jerusalem, Jerusalem, Israel; 4 Department of Psychology, University of Groningen, Groningen, The Netherlands

**Keywords:** Social movement, Social momentum, Intervention tournament, Psychology, Psychology, Sociology

## Abstract

Social protests can play a crucial role in shaping a nation’s direction. But what drives people to stay dedicated to a cause over time? This study was the first to implement a psychological intervention tournament (*N* = 1218) within the context of an ongoing social movement, testing the effectiveness of three psychological interventions in promoting sustained engagement in the pro-democracy protests in Israel in 2023, when citizens rallied against a proposed legal reform that threatened to weaken democracy. Our findings revealed that a momentum-based intervention effectively kept participants involved in the movement two and a half months post-exposure, outperforming morality-based and identity-based interventions. These results provide novel and valuable insights for sustaining social protests in pursuit of societal change.

**Supplementary Information:**

The online version contains supplementary material available at 10.1038/s41598-026-43735-x.

## Introduction

Social protests (Social protest is defined as a form of political expression that aims to bring about social or political change by influencing the public’s knowledge, attitudes, and behaviors, or an organization or institution’s policies (Shuman, Goldenberg, Saguy, Halperin, & van Zomeren, 2023.) can be a powerful mechanism for social change by reshaping social, political, and economic structures. For example, the Black Lives Matter movement’s continuing fight against police violence and systemic racism has effectively led to raised global awareness about racial inequality^1^. Similarly, the Fridays for Future movement, led by youth activists, has succeeded in drawing public attention to environmental issues, forcing both media and policymakers to engage with the issue^2,3^. However, most protests fail to achieve their goals, and even successful ones demand long struggles^4^. This raises a key question for those seeking social change - What drives people to stay dedicated to a cause over time?

The current study highlights perceived momentum (Perceived momentum is defined as the subjective feeling of being in a movement forward and getting closer to a goal (see Cohen-Eick at el.^10^).) as a key factor for sustaining the largest protest in Israel’s history in 2023. This protest started when the Israeli government attempted to pass legal reforms that would have weakened democracy in the country, and 20% of Israel’s population took to the streets in protest, demanding that the decision-makers stop the legislation. The protest lasted for nine consecutive months. In this situation, where maintaining mobilization is essential, we designed the first ever intervention tournament for protest mobilization over time, testing which psychological interventions are most effective in sustaining participation in the protest. We found that, compared to a control and two other interventions – i.e., identity- and morality-based interventions, the momentum-based intervention during this real-life protest campaign effectively maintained participation up to two and a half months after participants were exposed to the interventions. Recent years feature an unprecedented frequency, scope, and size of global mass protests^5^. In many of them, individuals participated in the protest on a weekly or even daily basis for long period of time. Since social protest can be effective by applying pressure to decision-makers^6,7^, sustained social protest can be even more effective as it applies pressure continuously and consistently^6^. This continuous aspect also gives the movement time to grow and mobilize additional people; as a broader portion of the population participates in the action, the more chances the protest will succeed in achieving its goals^4^. From the Civil Rights Movement in the U.S. (1950s–1960s) to the 2019–2022 Chilean protests, mass movements often feature a power in numbers that can force governments to make concessions or change course.

Nevertheless, participation in ongoing action entails a considerable and repeated effort on the protestors’ side without any guaranteed outcomes. According to Klandermans and Oegema^8^, to become a participant in a social movement, one must be motivated to overcome instrumental barriers to actual participation^8^. In this context, such barriers can include repeated time and travel costs, fatigue and burnout, fear of escalation or violence, competing work and family obligations, and uncertainty about whether continued participation remains consequential during pauses or negotiations. Similarly, CIVICUS, a global alliance of civil society organizations and activists dedicated to strengthening citizen action and civil society throughout the world, highlights the challenges faced by protestors in sustaining movements, such as maintaining motivation over time, ensuring safety from police brutality, and navigating legal and societal barriers, including potential government repression^9^. Scholars have also argued for important differences between sustained (versus momentary) social movements^10–14^. Thus, mobilizing people for one protest is one challenge, but keeping them engaged is quite another. Ongoing protest participation can also be conceptualized as a collective action dilemma^15^, because the benefits are non-excludable and individuals may be tempted to free-ride when their personal contribution feels negligible^16^. So, what can keep people engaged over time and prevent dropout?

A promising answer might be perceived momentum (Perceived momentum can be thought of as being similar to but also different from adjacent constructs, such dynamic norms (i.e., social norms that reflect how other people’s behavior and attitudes are perceived to be changing over time)^22,23^. Although both refer to the external and social environment, momentum encompasses more specific yet key elements that are not present in dynamic norms, such as a sense of progress toward a goal, gaining strength, and achieving success.),^17^. Adler and Adler^18^ define momentum as a force that motivates individuals toward a goal, either gradually or dramatically. Adler^19^ describes it as a dynamic state characterized by motion and success. Hence, we define psychological momentum in the context of social protest as “a sense of being in a movement forward and getting closer to the group’s goal.” Cohen-Eick and colleagues^10^ showed that perceived momentum is positively associated with sustained action, and Chenoweth and Belgioioso^6^ demonstrated that movements with greater momentum are likely to lead to more successful outcomes. This includes, for example, perceiving an increase in the number of demonstrators and the frequency of protest events^6^, a sense of progress towards the goal^20^, gaining perceived strength and achieving success^21^. The focus is hence on the perceived ongoing movement towards increasing achievements and participation, rather than just looking back at past accomplishments and the number of people involved. For those involved in sustained action, what may be unique about perceived momentum is that it keeps participants in (rather than motivating them even more)^22,23^.

If we borrow the concept of momentum from Newton’s first law of physics^24^ and compare a social movement to an object’s movement, we can see that an initial push is required to set the object into motion. Once in motion, and assuming there is no friction, the object would continue to move forward indefinitely. However, in reality, various forms of friction exist that can impede movement. In the social reality of social movements, core motivations for social protest such as identity and morality^25^ may offer the initial push needed to start a movement. Identity refers to individuals’ sense of belonging to a group and the motivation to act collectively on its behalf, that is, ‘who we are.’ Morality refers to motivations rooted in deeply held values and moral convictions about right and wrong, that is, ‘what we stand for.’ While both identity and morality were shown to predict protests^25–27^, a recent study suggests they may be less suited to sustaining repeated participation^10^. We therefore propose that perceived momentum can help sustain engagement by counteracting the unique challenges associated with sustained engagement. Momentum interventions might uniquely serve to overcome these frictions, allowing the movement to continue forward without needing an additional motivational push.

We designed the current study to evaluate this proposal. Specifically, we tested a momentum-based intervention during a real-life protest setting to empirically evaluate its efficacy in maintaining participation against a control condition, and against morality- and identity-based interventions. The momentum-based intervention was therefore developed to increase one’s sense of progress. While building on previous work^6,20,21^, the main design guideline was to tell the public that something significant is happening, that it is growing, making noise, and on its way to its goals. The call for action was to join this big movement as it moves forward. The democratic-identity and moral-harm interventions were based on established theoretical constructs shown to motivate participation in social movements: identity^23,25^ and moral conviction^22,25^. These frameworks have been widely used to predict collective action (in correlational, longitudinal, and experimental studies^23,28^ and thus serve as theoretically grounded comparison conditions. In the current study, we develop interventions for both morality and identity, and its design enables us to distinguish whether the momentum intervention’s effect might be stronger than those of other mobilization messages derived from the literature^25^. Identity refers to individuals’ sense of belonging to a group and the motivation to act collectively on their behalf, i.e., “who we are”^25,29^. Therefore, the intervention focused on an identity as Democrats (The researchers would like to clarify that the term “Democrats” pertains specifically to supporters of democratic governance and should not be confused with the names of political parties.) and the need to defend this shared identity, as if the reform would pass, it would be hard to identify as one in a non-democratic state. Morality refers to individuals’ motivations to act that stem from deeply held values or moral convictions guiding what they see as right or wrong, simply put, “what we stand for”^25,29^. In the current context, the moral harm intervention highlighted core democratic values and moral beliefs that the reform will impact, should it pass. Participants in the control condition were not exposed to any mobilization message as part of the experiment and were unaware that other participants had received one. This group, therefore, served as a baseline for behavioral comparisons, acting as a conservative control.

Since momentum should be particularly relevant to participation over time in social protest, our main hypothesis was theoretically derived and specified prior to data collection: that the momentum-based intervention would uniquely sustain protest participation over time - that is, maintain participation across waves - compared to the moral-harm and democratic-identity interventions. Analyses comparing the other conditions are therefore considered exploratory. This hypothesis was tested using the intervention tournament protocol^30^, comparing participation rates over time across the four experimental conditions.

**Fig. 1:**
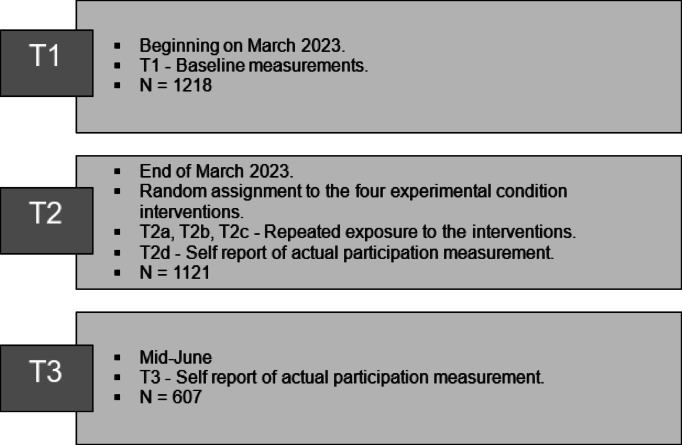
Study waves and surveys.

We conducted a three-time-point longitudinal study, beginning at the end of February to the beginning of March 2023 as the protest began, for baseline measurement – T1. Then, at the end of March, all participants were randomly assigned to one of four conditions. Within a week time frame, four waves were held: three waves of the interventions, in which participants were exposed to the same intervention repetitively for three times; and one wave of measuring actual participation following the interventions. Specifically, wave T2a consisted of exposure to mobilization messages as videos and as posters, wave T2b included exposure to mobilization messages as posters only, and wave T2c was comprised of exposure to mobilization messages as videos for the second time. The fourth wave, T2d, was conducted by the end of the week, in which participants were asked about their participation in the large demonstration that Saturday night, the dependent variable. Lastly, the sixth wave, T3, was conducted two and a half months after T2 in which, participants were asked about the dependent variable again, their participation in the demonstration that week (See Fig. 1).

It is important to note that T1 served solely as a baseline measurement. The interventions were designed in the brief period between T1 and T2, during which the protest movement was rapidly intensifying. Because the opportunity to develop and immediately deploy theory-grounded interventions in this real-world context is extremely time-sensitive, we prioritized it over pre-registration. We developed and conducted this unique intervention tournament in collaboration with the social movement, which allowed us to co-develop a campaign based on scientific research. All interventions consisted of mobilization messages such as videos and posters to which the participants were exposed (see Supplementary Materials Appendices 1–2 for the full intervention scripts).

## Results

A total of 1218 participants, randomly sampled from the mainstream Jewish Israeli public were included in the analysis, of which 298 (24.5%) were assigned to the momentum-based intervention, 303 (24.9%) were assigned to the democracy identification-based intervention, 306 (25.2%) were assigned to the moral harm-based intervention, and 311 (26%) were assigned to the control condition (conservative control). Once a participant was assigned to a condition intervention, they were exposed to materials from the same campaign for three times, as detailed above. While the campaign was presented to participants three times, not all participants viewed the campaign each time it was available (meaning they received a link but did not open it), similar to real-world campaigns where exposure can vary among target audiences. Given this, participants’ exposure to the interventions was distributed as follows: 672 participants (55.2%) observed all three exposures of their assigned intervention, 354 participants (29.8%) observed two out of the three exposures of their assigned intervention, and 192 participants (15.1%) observed one out of the three exposures of their assigned intervention (a or b or c). There were no significant differences in mean exposure between conditions (*F*(3, 1214) = .15, *p* = .93), categorical distribution of exposure levels differed across conditions (*χ²*(6, *N* = 1218) = 18.031, *p* = .006; see Table S8). Moreover, 595 (48.89%) participants had past protest experience, while 622 (51.11%) did not prior to the study. Demographic characteristics by condition are reported in Supplementary Table S7. In a longitudinal study during turbulent real world events dropout bias and complexity is an inevitable challenge, so we conducted dropout analysis and employed control variables.

There were no significant differences between the four conditions at times T2d and T3 for political ideology, age, and number of exposures (one-way ANOVAs; all *p* > .05; see Supplementary Tables S9–S10 for balance checks, see Supplementary Tables S1–S4 for attrition analysis; see Supplementary Table S8 for exposure checks). In general participants who completed all waves were older (*M* = 44.58) than those who did not (M = 42.63; *t*(≈ 1215) = 2.30, *p* = .021) and tended to left-wing ideology (*M* = 4.01) in comparison to participants who began the study and did not complete it (*M* = 4.2; *t*(≈ 1215) = − 2.54, *p* = .011). Given the political context of a protest against a right-wing government, it could be assumed that participants identifying as left-leaning or centrist would be more likely to sympathize with the cause, which may explain their higher baseline participation rates^31,32^. Analyses without covariates did not substantively alter the pattern of results, see ‘Robustness Analyses Without Covariates’ section in supplementary materials. Additional analyses, including attrition checks, dropout predictors, and robustness analyses, are provided in the Supplementary Materials (Tables S1–S11).

### Interaction effects in logistic regression analysis

**Fig. 2:**
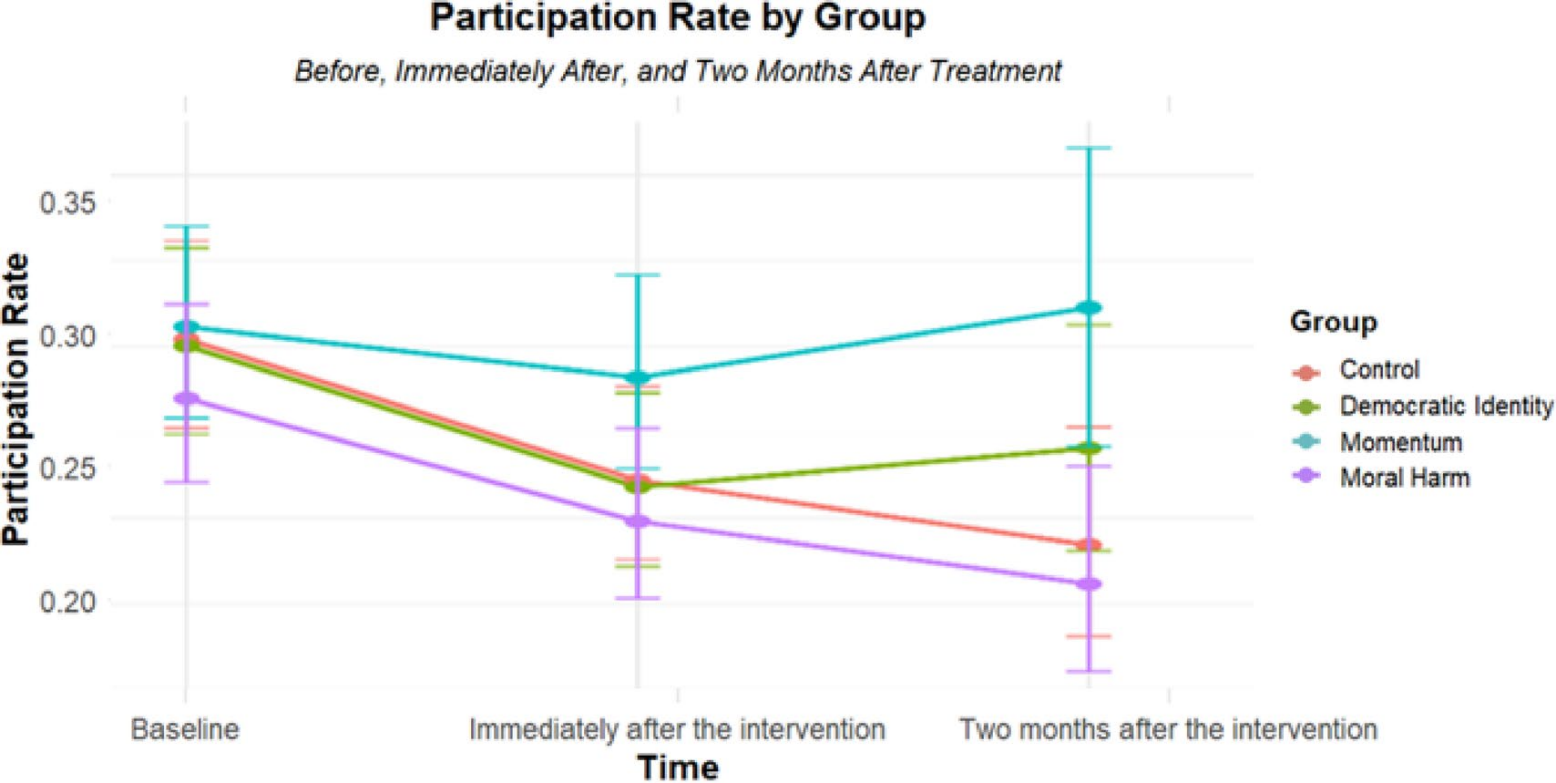
Participation rate by wave and group. The graph visualizes the changing participation percentages over time across three experiment waves, with each color representing a different experimental condition. Points indicate the percentage of participation, while bars represent 95% confidence intervals. Participation rates refer to participation in the protests.

A logistic regression analysis was conducted to examine the effect of the experimental conditions over time on protest participation. The model included the effects of time (as a factor with three levels, dummy coded with T1 as the reference category: T1, T2, T3), experimental condition (Momentum, Moral Harm, Democratic Identity, Control; dummy coded with control as the reference category), and their interactions, while controlling for number of intervention exposures, age, and political ideology. The results are summarized in Table 1; Fig. 2. To address the missing data while maintaining the high N number, the logistic regression was applied in a between-subjects framework. Alternative model specifications, reported in the Supplementary Materials, yielded similar conclusions. We verified standard assumptions for logistic regression (e.g., multicollinearity and model fit); additional diagnostic information is reported in the Supplementary Materials.

**Table 1:** Logistic regression results predicting protest participation with interaction effects.

Predictor	OR	SE	z	95% CI	*P*
Intercept	0.0139	0.281	-15.189	0.008 – 0.024	< 0.001***
Time 2	0.307	0.195	-6.064	0.210 – 0.450	< 0.001***
Time 3	0.242	0.243	-5.830	0.150 – 0.390	< 0.001***
Democratic Identity-based intervention	1.014	0.181	0.077	0.712–1.449	0.939
Momentum-based intervention	1.071	0.181	0.381	0.751–1.527	0.704
Moral Harm-based intervention	1.015	0.180	0.085	0.713–1.444	0.932
Exposure	1.019	0.062	0.302	0.902–1.152	0.763
Age	1.021	0.003	7.160	1.014–1.027	< 0.001***
Political ideology	2.179	0.040	19.337	2.047–2.315	< 0.001***
Time 2 × democratic identity	0.962	0.276	-0.143	0.602–1.652	0.886
Time 3 × democratic identity	1.399	0.336	1.000	0.724–2.704	0.317
Time 2 × Momentum	1.296	0.273	0.946	0.759–2.214	0.344
Time 3 × Momentum	2.010	0.341	2.047	1.030–3.922	0.041*
Time 2 × Moral harm	0.847	0.278	-0.597	0.492–1.461	0.551
Time 3 × Moral harm	0.830	0.356	-0.522	0.413–1.668	0.602

*p < .05; **p < .01; ***p < .001. All OR values have been transformed exponentially. Control was the reference category for condition, and T1 was the reference category for time. Time was dummy-coded (T2, T3 vs. T1) and condition was dummy-coded (Democratic identity, Momentum, Moral harm vs. Control). Covariates (exposure count, age, and political ideology) were entered as continuous predictors.

In the overall model, participation declined significantly over time across all participants (*χ²*(2) = 73.6, *p* < .001) indicating that, on average, protest participation decreased at the later measurements. Compared to T1 (the reference category), protest participation decreased significantly at T2 (*OR e*^(− 1.182) ≈ .307, *SE* = .195, *z* = -6.064, *p* < .001) and T3 (*OR e*^(− 1.418) ≈ .242, *SE* = 0.243, *z* = -5.830, *p* < .001). This indicates that without any intervention there is decline in protest participation over time. Among the control variables, age (*OR e*^( .021) ≈ 1.021, *SE* = .003, *z* = 7.160, *p* < .001) and political ideology (*OR e*^( .779) ≈ 2.179, *SE* = .040, *z* = 19.337, *p* < .001) were significant positive predictors of protest participation among the control condition. This suggests that older participants and those identifying as more left-leaning were more likely to participate in protests without intervention compared to younger and more right-leaning participants.

The critical finding pertains to the interaction effects between time and the Momentum-based intervention. The interaction between T3 and the Momentum-based intervention was significant (*OR* e^( .698) ≈ 2.010, *SE* = .341, *z* = 2.047, *p* = .041), indicating that, at T3, the Momentum intervention had a positive effect on protest participation. No other intervention recorded higher participation rates when compared to the control group or over time.

The significant positive interaction between T3 and the Momentum-based intervention suggests that participants in the Momentum-based intervention were more likely to participate in protests at T3 compared to those in the Control condition, beyond the effects of time and condition. No other interactions were significant, indicating that the effect of the other experimental conditions on protest participation did not differ significantly over time compared to the Control condition.

### Pairwise comparisons

**Fig. 3:**
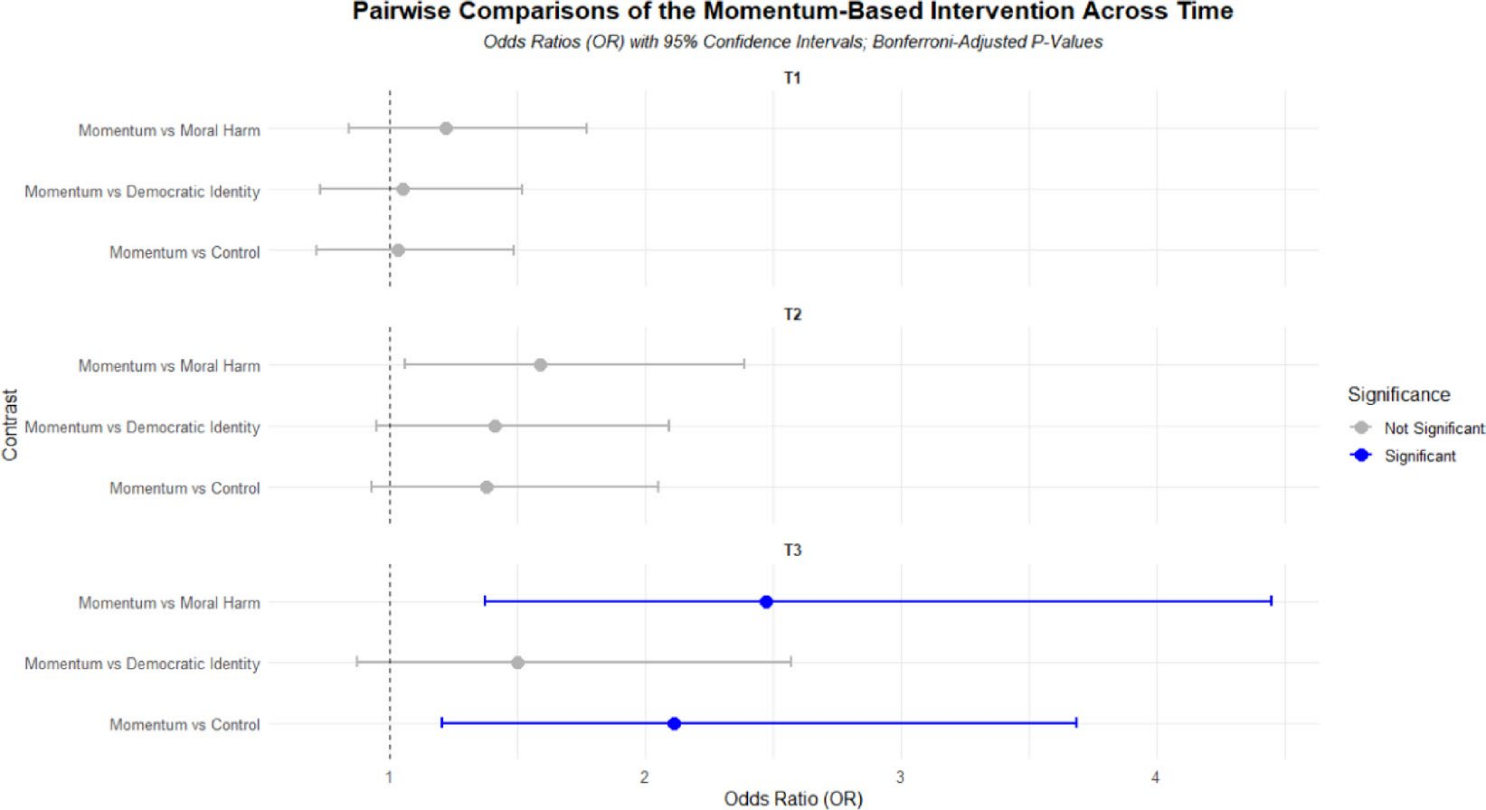
Pairwise comparisons of the ‘momentum’-based intervention vs. other conditions at each time point. This graph displays the pairwise comparisons of the Momentum-based intervention relative to other conditions (Control, Democratic Identity, and Moral Harm) at three different time points (T1, T2, and T3). Odds ratios (OR) represent the likelihood of protest participation in the Momentum condition compared to the other conditions at each time point. Error bars represent 95% confidence intervals. A dashed vertical line at OR = 1 represents the threshold for no effect. Comparisons that remain statistically significant after Bonferroni adjustment are highlighted in blue, while non-significant comparisons are shown in gray.

Pairwise comparisons between the momentum-based intervention and other conditions at each time point were conducted using odds ratios (OR) with Bonferroni-adjusted p-values. The results are summarized in Table 2; Fig. 3.

**Table 2:** Pairwise comparisons of the ‘momentum’-based intervention vs. other conditions at each time point.

Time	Contrast	OR	SE	z	95% CI	*p*
1	Momentum vs. Control	1.07	0.194	0.381	0.728–1.573	1.000
1	Momentum vs. Democratic Identity	1.06	0.194	0.300	0.721–1.559	1.000
1	Momentum vs. Moral Harm	1.06	0.192	0.295	0.725 − 1.550	1.000
2	Momentum vs. Control	1.39	0.284	1.602	0.793–2.438	0.327
2	Momentum vs. Democratic Identity	1.42	0.292	1.722	0.805–2.503	0.255
2	Momentum vs. Moral Harm	1.61	0.338	2.285	0.834–3.102	0.067
3	Momentum vs. Control	2.15	0.622	2.655	0.637–7.258	0.024
3	Momentum vs. Democratic Identity	1.52	0.424	1.494	0.661–3.499	0.406
3	Momentum vs. Moral Harm	2.55	0.777	3.081	0.513–12.705	0.006

*OR* = odds ratio; *SE* = standard error; *z* = z-score; *p*-values are Bonferroni-adjusted.

At T3, the momentum-based intervention had significantly higher odds of protest participation compared to the control condition (*OR* = 2.15, *SE* = .622, *z* = 2.66, *p =* .024). This indicates that participants in the momentum-based intervention were more than twice as likely to participate in protests as those in the control condition at T3. Similarly, the momentum-based intervention showed significantly higher odds compared to the moral harm-based intervention (*OR* = 2.55, *SE* = .78, *z* = 3.08, *p=*.006). This indicates that, at T3, the momentum-based message was associated with higher sustained participation than the moral-harm message. No significant differences were found between the momentum-based intervention and the democratic identity-based intervention at T3 (*OR* = 1.52, *SE* = .42, *z* = 1.49, *p=*.406), suggesting that these two messages produced comparable participation levels at follow-up. At T1 and T2, there were no significant differences between the momentum-based intervention and other conditions after adjusting for multiple comparisons (all *p* > .05; see Table 2 for full results).

## Discussion

We studied momentum in sustaining actual participation in ongoing real-life social protests up to two and a half months after exposure to the intervention, set within the context of the 2023 Israeli pro-democracy movement. We found that participants in the momentum-based intervention were approximately twice as likely to continue participating in the protests compared to the control group after two and a half months, demonstrating the power of perceived forward movement and goal progression in sustaining long-term engagement. This delayed pattern (i.e., the momentum-based condition did not differ from the other conditions at T2, the difference emerged at T3) may reflect how perceptions of collective progress accumulate over time rather than producing an immediate behavioral response, which is consistent with prior work suggesting that continued engagement can depend on the accumulation of perceptions over time rather than on a single immediate trigger^10,13,33^, making delayed divergence across conditions plausible in longitudinal protest contexts. It is also possible that contextual developments contributed to the strengthening of this perception. Specifically, during the period between T2 and T3, the protest achieved a major victory. It was agreed to halt the legislation and initiate discussions between the opposition and the coalition in Israel to reach a compromise on the proposed legal reform. This achievement was credited to the protest and had the potential to build momentum. However, some voices emerged, calling for the protest to end since its goal had been achieved. They argued that the compromise conversations should proceed quietly, which could have significantly reduced the general sense of momentum. These events may have reinforced participants’ already existing momentum sense, thereby amplifying the psychological experience of momentum among those exposed to the momentum-based message (Because we did not measure participants’ perceptions of these political developments directly, we present this as a plausible contextual influence rather than as an inference from our data).

Additionally, the decline in participation over time, evident in the effect of time and the slope of the control conditions, suggests that maintaining involvement in this context indeed was challenging. Against this backdrop, these findings highlight the crucial role of perceived momentum in keeping participants engaged in prolonged social movements^6,10^, aligning with earlier research suggesting that the perception of progress can significantly motivate individuals to remain committed to a cause^17^. Indeed, a momentum-based intervention may help overcome external barriers (e.g., fatigue, competing obligations, safety concerns) that would otherwise reduce participation, allowing the movement to continue without an additional motivational push. As we did not directly measure potential barriers, we offer a possible interpretation that perceived progress may make continued participation feel more worthwhile when success seems within reach.

Although the difference between the momentum and democratic-identity interventions was not statistically significant, this equivalence in observed behavioral outcomes is itself theoretically informative. It suggests that, while only the momentum condition (unlike the identity condition) showed a significant increase compared to the control group, there might be a shared process. Because we did not directly measure mediating psychological processes in this study, we offer the following as a speculative interpretation rather than a tested mechanism. One possible interpretation is that momentum messages partially incorporate elements of identity (e.g., portraying the group as moving forward together) yet go beyond identification by emphasizing ongoing progress and success. This additional focus on perceived advancement may explain why the momentum intervention uniquely translated into sustained behavioral engagement over time. Moreover, the two messages differ in their motivational focus: the identity message highlights the problem — a threat to a valued democratic identity — whereas the momentum message highlights an emerging solution, conveying that the movement is already advancing and gaining strength. This forward-looking emphasis on potential solutions may help individuals focus on solutions rather than dwell on problems, which may keep the participants engaged over time with the solution, the protest. Understanding these potential process-level commonalities or differences remains an important direction for future research.

At the individual level, a major challenge underlying social protest participation (the collective action dilemma^15^ is the free-rider behavior^16^. When the unique contribution of each participant seems negligible (e.g., “A single person’s participation in a demonstration has no impact on the visibility of the protest”) or when there is a lack of faith that others will also participate (e.g., “It’s not fair that one should go to the trouble of demonstrating for everyone while others stay home comfortably”), there is an incentive to free-ride and not participate. It is possible that perceiving momentum, that is, perceiving that the movement is growing and making progress, may be a solution to these problems. Momentum signals may indicate that the action has an impact and is therefore worth pursuing, as well as signaling that others are already participating in it. Additionally, the perception of effectiveness may strengthen feelings of empowerment and significance, thereby reinforcing the sense that one’s participation contributes to a meaningful collective goal. Such experiences of significance gain and perceived impact are known to predict participation^25^. Alternatively, it is possible that during a prolonged and emotionally charged protest context, appeals to democratic identity or moral concern may have already been salient in public discourse, thereby limiting the added value of these interventions.

Despite the promising results of this unique intervention tournament, it has certain limitations as it is conducted during a real-life event. There is the trade-off between effectively capturing phenomena as they unfold in the real world with the opportunity to influence them in real-time and the ability to control all possible variables. Additionally, before the T3 measurement, protest leaders utilized our preliminary findings (as the collaboration was mentioned before), which likely caused some overlap between the conditions (During this period in Israel, extensive mobilization efforts aimed to boost protest participation. Consequently, many participants across all four conditions likely encountered varying appeals from different sources, potentially leading to overlapping influences. During the collaboration with protest organizers, our feedback focused on emphasizing momentum-based framing to highlight perceived collective progress. If any overlap in messaging occurred in practice, it was likely momentum appeals to participants in the other frames, rather than the other way around). Accordingly, we interpret effect sizes cautiously and recommend replication under conditions with greater message separation. Other conditions might have also benefited from the momentum intervention implemented in the movements general messaging. Additionally, the study was conducted within a specific cultural and political context — Israel during the 2023 pro-democratic movement. As such, the findings relevance to other settings should be considered with an awareness of the specific circumstances surrounding these events. Future studies should explore how momentum interventions might operate in different cultural and political settings to determine their broader applicability across various social movements.

As far as we know, this is the first-ever psychological intervention tournament related to sustained participation in social movements. Moreover, it features real-world interventions aimed at changing behavior outside of lab settings. Beyond the potential of momentum interventions, there is a fundamental opportunity for academics and practitioners to develop psychological interventions that can have a real-world impact. In times where democratic principles are believed to be at stake, it is crucial to create tools that empower individuals to become active citizens and advocate for their democratic values. We hope this study will pave the way for additional intervention research to promote civic engagement and drive social change.

## Materials and methods

Participants responded to all measures on a 6-point Likert-type scale, where responses ranged from 1 (Not at all) to 6 (strongly agree) unless otherwise specified. There were additional variables measured during the T1 survey that were not part of the study and, therefore, were excluded from the analysis, such as different identity items, attitude towards moral foundation, attitude towards democracy principles, and beliefs about the future of the protest. This study’s OSF project reports the complete set of measures (https://osf.io/xmkpf).

### Gender and age

One item each assessed gender and age. Participants were asked to indicate their gender and age. Age was reported in years. Gender was self-reported (male, female, or other/unknown).

### Political ideology

Only one item on the T1 questionnaire assessed political ideology, ranging from 1 (Extreme right) to 7 (Extreme left). Values 1–3 were combined to form Rightists, 4 became Centrists, and 5–7 were grouped as Leftists.

### Actual action participation

One item measured actual participation in an action. In T1, participants were asked whether they participated in the protest against the legal reform. On T2d, participants were asked whether they participated in one of the demonstrations against the legal reform on a specific Saturday night, on a scale of Yes (with an option to mark that they are on their way or currently in a demonstration for those filled out the survey Saturday night) and No (with an option to mark if they planned in advance to participate or not). In T3, participants were asked whether they participated in one of the demonstrations against the legal reform during that week.

### The video interventions

One video out of three was presented to participants on waves T2a and T2c. Participants needed to complete a video and sound checks to proceed to the intervention. The instruction was: “On the next screen, a randomly selected video will appear from several campaigns that are running now on the topic of the protest against the legal reform. We will ask you to watch the video and then answer questions. Among other things, we would like to know what you think about the video you watched”. The momentum intervention was grounded in previous research. Drawing on the work of Chenoweth and Belgioioso (2019), we highlighted the increase in both the number of participants and the frequency of protests. Additionally, based on the findings of Cohen-Eick and colleagues (2023), we focused on the process of approaching forward and getting closer to the protest’s goals. The democracy identity video emphasizes shared democratic values that define the identity, that democratic identity unites the protesters, that participating in protests is defending democracy, and that holding a democratic identity means participating in the protest. The moral harm video emphasizes how the legal reform would violate the moral foundations (which include harm, fairness, freedom from oppression, loyalty, authority, and purity). Given that the judicial reform is generally seen as violating all six, we have decided to highlight at least one individualizing foundation (harm) and at least one binding foundation (loyalty, purity) to help it appeal to leftists and center/rightists. For the complete video scripts, see Supplementary Materials Appendix 1. Content validity results are reported in Supplementary Table S6.

### The poster interventions

One poster out of three was presented to participants on waves T2a and T2b. The instruction was: “On the next screen, one ad will appear out of several ads that are running right now on the protest against the legal reform. After the presentation, we will ask you to answer some questions”. The momentum poster emphasizes how protests have grown and gained the momentum they need to reach their goals. The democracy identity poster emphasizes shared democratic values that define the identity and that holding a democratic identity means participating in the protest. The moral harm poster emphasizes how the legal reform would violate the moral foundations: harm, fairness, freedom from oppression, loyalty, authority, and purity, and that there is no such thing as compromise in these values. For the full text of the posters, see Supplementary Materials Appendix 2.

## Supplementary Information

Below is the link to the electronic supplementary material.

Supplementary Material 1

## Data Availability

The datasets, materials, and analysis code used in this study are publicly available on the Open Science Framework (OSF) - https://osf.io/xmkpf.
