# Supplementary material for: A novel momentum-based intervention sustains real-life participation in a social movement
Source: Sci Rep. 2026 Mar 17;16:13855. doi: 10.1038/s41598-026-43735-x (PMC13129044; doi:10.1038/s41598-026-43735-x)
Supplement: Supplementary file 1 — Supplementary Material 1 [file 41598_2026_43735_MOESM1_ESM.docx]

**Supplementary Materials for**

A Novel Momentum-Based Intervention Sustains Real-Life Participation in a Social Movement

**This PDF file includes:**

Supplementary text

Tables S1 to S11

Figure S1

Appendix 1 to 2

**Reading guide**

This Supplementary Materials document provides additional analyses, robustness checks, and materials that support the main manuscript while keeping the Results section concise. The document is organized in the same order that the analyses are referenced in the manuscript, so readers can locate Supplementary Materials quickly. Below, we briefly explain the purpose of each section and why each analysis was conducted.

1. **Attrition analyses (Tables S1–S4)**

Due to the fact that this study was conducted during an ongoing real-world protest context, attrition across waves was expected. We therefore examined whether dropout differed by experimental condition and whether dropout was systematically related to participant characteristics. These analyses helped evaluate potential attrition bias and motivated our decision to include key covariates (age, political ideology, and exposure) in the main models. Specifically:

- Table S1 summarizes dropout rates by condition to document condition-level attrition.
- Table S2 models predictors of dropout to identify which variables were associated with missingness and to assess whether dropout patterns differed by condition when controlling for covariates.
- Tables S3–S4 compare dropouts and completers within each condition on continuous and categorical variables to provide a transparent check of potential selective attrition.

1. **Completer-only robustness analysis (Table S5)**

To evaluate whether the main longitudinal conclusions depended on including participants with incomplete follow-up data, we re-estimated the primary model using only participants who completed T3. This analysis provided a robustness check that addressed concerns that differential attrition (especially in the momentum condition) might drive the main results.

1. **Content validity of intervention materials (Table S6)**

To document that the intervention messages captured their intended psychological frames (momentum, democratic identity, moral harm), we report expert ratings of the intervention content. This analysis was included to provide evidence that the manipulations aligned with their theoretical constructs.

1. **Exposure distribution checks (Table S7)**

In real-world-style campaigns, exposure varies because participants do not always view materials when available. We therefore examined exposure patterns across conditions (both as mean exposure and as exposure categories).

1. **Demographic descriptives and balance checks (Tables S8–S11)**

To facilitate interpretation and for clearer reporting, we provide:

- Descriptive characteristics for the full sample by condition (Table S8).
- Balance checks among T2d respondents and among T3 respondents (Tables S9–S10), testing whether those who responded at later waves differed across conditions on key variables (age, ideology, exposure, income). These checks clarify whether differential response at later waves could confound condition comparisons.
- A baseline equivalence check for past protest participation (Table S11), showing that conditions did not differ in prior participation before the interventions were delivered.

1. **Additional robustness models (Figure S1)**

To complement the primary between-subjects logistic regression approach, we report an additional longitudinal model that explicitly accounts for repeated measures within participants (GLMM). This analysis was conducted to verify that the pattern of results was consistent under an alternative modeling framework that accounts for within-person dependence and missingness under standard assumptions.

1. **Appendices (Appendices 1–2)**

Appendices include the full intervention scripts and poster texts (English translations), to ensure transparency and reproducibility and to allow readers to evaluate the content of the messages directly.

Supplementary Materials Text

**Method**

***Participants***

One thousand two hundred eighteen participants were recruited (M_age_ = 42.76, 51% female, 33% centrist, 36% leftist, 31% rightist.). The inclusion criteria were for the participants to be at least 18 years old. The recruitment process was conducted by a survey company.

For the sixth questionnaire (T3), 607 participants were returned from the sample of participants who had completed the previous surveys. There were no differences in dropout rates except in age, political ideology, and the number of intervention exposures. Namely, there were no differences in meaningful socio-demographic measures between the participants who completed all waves of the study and participants who did not fill out the sixth survey. Age and political ideology significantly differed (see Attrition analyses, Table S3; see also the multivariable dropout model in Table S2). Participants who filled the last survey were older (*M* = 44.58) than those who did not (*M* = 42.63) and tended to left-wing ideology (*M* = 4.01) in comparison to participants who did not complete the last survey (*M* = 4.2). Given the political context of a protest against a right-wing government, it could be assumed that participants identifying as left-leaning or centrist would be more likely to sympathize with the cause, which may explain their higher baseline participation rates (Berenson & Halperin, 2021; Israel Democracy Institute, 2023). However, we control for both age and political ideology in the analysis. See Attrition analyses (Tables S1–S2) for dropout rates by condition and the multivariable dropout model..

In addition, there were no differences that could influence the results between the experimental conditions due to dropout over time, according to logistic regression analyses summarized in Table S2. A one-way ANOVA model was conducted to predict differences based on four experimental conditions. The result indicates no significant differences between the four conditions in time T2d and T3 for political ideology, age, number of exposures to the interventions, and income. A logistic regression showed no significant differences in participation in the protest prior to our study.

Gender differences across conditions were tested using logistic regression with the control condition as the reference category. At T2d, compared with the control group (56% female), the moral-harm group (47% female) had lower odds of female respondents (*B* = -0.35, *SE* = 0.17, *z* = -2.05, *p* = .040), and the democratic-identity group (48% female) also had lower odds (*B* = -.34, *SE* = .17, *z* = -2.01, *p* = .045); the momentum group did not differ from control (52% female; *B* = -.15, *SE* = 0.17, *z* = -.91, *p* = .364). At T3, compared with the control group (57% female), the moral-harm group (43% female) again had lower odds of female respondents (*B* = -.55, *SE* = 0.23, *z* = -2.37, *p* = .018), whereas the democratic-identity group (50% female; *p* = .196) and the momentum group (51% female; *p* = .291) did not differ from control.

***Procedure***

The study consisted of six surveys. The first survey, T1, was conducted for baseline at the end of February and the beginning of March 2023 as the protest began. Then, at the end of March, within a week, the following three waves were held^[[1]](#footnote-1)^: wave T2a - exposure to mobilization messages as videos and posters, wave T2b - Exposure to mobilization messages as posters, and wave T2c - exposure to mobilization messages as video for the second time. The fifth wave, T2d, was conducted by the end of the week, in which participants were asked about their participation in the big demonstration that Saturday night. Participants who did not complete their questionnaires on time were asked about the big protest of the following Saturday night demonstration. Lastly, the sixth wave, T3, was conducted two and a half months after T2 (See Fig 1 in main text).

All materials were co-developed with organizers of the pro-democracy movement to ensure ecological validity and realism. Consequently, minor stylistic differences between the messages (e.g., occasional capitalization or varying levels of direct address) reflect authentic mobilization styles rather than systematic differences across experimental conditions. The intervention materials were originally delivered in Hebrew. Because Hebrew does not distinguish between uppercase and lowercase letters, the capitalization in the English translation was added only to approximate visual emphases, such as color contrasts and font size, in the original materials. The original Hebrew versions of all videos and posters are available in the study’s OSF repository - <https://osf.io/xmkpf>.

The three mobilization messages were developed iteratively and reviewed by both researchers and protest advisers to ensure that each message clearly reflected its intended frame, supporting the conceptual validity of the manipulation. To ensure the integrity of our findings, we conducted a validity analysis of the interventions based on an experts survey.

**Content Validity**

Content validity was assessed using expert ratings (N = 19) on a 1–6 scale. Following Polit and Beck’s (2006) guidelines for Likert-type scales, ratings of 4–6 were coded as indicating item relevance. Item-level CVI (I-CVI) values ranged from .58 to .95 across the nine items (Table S6). The identity items demonstrated strong content validity (I-CVI range = .74–.95; S-CVI/Ave = .88). The moral-harm items showed acceptable to excellent content validity (I-CVI range = .68–.95; S-CVI/Ave = .82). The momentum items also demonstrated good content validity (I-CVI range = .58–.95; S-CVI/Ave = .82), with one item rated as less representative than the others.

To further examine consistency among experts regarding the relative ranking of items within each construct, Kendall’s coefficient of concordance (W) was computed. Agreement was modest for the identity (*W* = .15, *p* = .062) and moral-harm items (*W* = .16, *p* = .049), indicating that experts generally viewed the three items within each construct as similarly representative. In contrast, agreement was higher for the momentum items (*W* = .44, *p* < .001), reflecting clearer differentiation among the items within this manipulation.

Overall, the CVI and Kendall’s W results jointly indicate that all three manipulations exhibit good content validity and appropriately represent their intended psychological constructs.

**Results**

Data were analyzed using logistic regression with interaction terms between time and treatment condition. Despite unequal numbers of observations at each time point, the analyses included all available data to maximize the sample use. The logistic regression was applied in a between-subjects framework to address the missing data. The logistic regression model appropriately adjusts for unequal group sizes, and estimated marginal means were calculated using the R emmeans package, which accounts for each group’s covariates and sample sizes.

The analyses followed the recommended intervention tournament protocol (Hameiri & Moore-Berg, 2022), in which all intervention arms are first compared to a shared control group before conducting direct pairwise contrasts between interventions. This design ensures consistent baselines, maximizes interpretability, and allows clear identification of the most effective intervention. Logistic regression models were therefore estimated using the control condition as the reference category, with Bonferroni-adjusted pairwise comparisons among intervention arms.

**Sensitivity Analysis**

Because opportunities to study large-scale social movements in real time are rare, the study was not pre-registered, and no a priori power analysis was conducted. To complement the reported logistic regression results, we conducted a post hoc sensitivity analysis to estimate the minimal effect size that the study design could reliably detect. The analysis was based on the observed participation rate in the control condition at T3 (*p* = .175; Table S5) and the condition-specific sample sizes at T3 derived from the attrition rates reported in Table S1 (Control ≈ 187, Momentum ≈ 146, Democratic Identity ≈ 174, Moral Harm ≈ 164). Assuming a two-tailed test with α = .05 and power = .80, the study was sensitive to detect odds ratios (OR) of approximately 2.27 (Momentum vs. Control), 2.06 (Democratic Identity vs. Control), and 2.13 (Moral Harm vs. Control). When applying a Bonferroni correction for three pairwise comparisons (α ≈ .0167), the corresponding detectable ORs were 2.55, 2.29, and 2.37, respectively. Analyses were conducted using the NormalIndPower function from the statsmodels package (v0.14.0) in Python.

These results indicate that the study was sufficiently powered to detect medium-to-large effects, which are consistent with typical effect sizes observed in real-world field interventions (e.g., Nickow, Oreopoulos, & Quan, 2024; Singla et al., 2017). While smaller effects may have gone undetected, the design was adequately sensitive to identify substantively meaningful behavioral differences between intervention conditions.

**Logistic regression models**

To retain the full available sample and handle missingness without resorting to listwise deletion, we applied a logistic regression model in a between-subjects framework, using all available observations. This strategy aligns with recommendations by Schafer and Graham (2002) and Graham (2009) to use likelihood-based methods that incorporate all available data rather than discarding incomplete cases. Logistic regression models are well-suited for between-subjects designs and remain robust even when group sizes are unequal (King & Zeng, 2001). Estimated marginal means (EMMs) were then computed using the emmeans package in R (Lenth, 2025), which adjusts for covariates and differences in group sizes, providing covariate-adjusted mean estimates for each group.

We examined key assumptions of the longitudinal logistic regression model. Variance inflation factors indicated no problematic multicollinearity, with GVIF^(1/(2·df)) values ranging from 1.00 to 2.00 for all predictors and their interaction. A Hosmer–Lemeshow goodness-of-fit test suggested adequate model fit, *χ²*(8) = 13.00, *p* = .10. Visual inspection of Pearson and deviance residual plots revealed no strong systematic patterns or heteroscedasticity, and the Q-Q plot of standardized deviance residuals showed only minor deviations from normality. Cook’s distances were all smaller than .01 (maximum *D* = .004), indicating that no single observation exerted undue influence on the estimates. Taken together, these diagnostics support the adequacy of the logistic regression model for the present data.

**Robustness Analyses Without Covariates**

To evaluate the robustness of our findings regarding the inclusion of covariates, we re-ran the primary logistic regression analyses excluding covariates (i.e., exposure, age, and political ideology). The model included time (T1–T3), experimental condition (Control, Democratic Identity, Momentum, Moral Harm), and their interaction as predictors of protest participation. The results were consistent with the main analyses that included covariates. Overall, the likelihood of participation decreased over time, *χ²*(2) ≈ 17.1, *p* < .001. At T2, participants were significantly less likely to report participation than at baseline (*OR* = .70, 95% *CI* [.49, .99], *p* = .045), and this decline continued at T3 (*OR* = .51, 95% *CI* [.33, .79], *p* = .003). Predicted probabilities from the model showed that participation rates declined from approximately 34% at baseline to 26% at T2 and 21% at T3 in the control condition. The momentum condition showed a higher predicted probability at the final measurement (34%) compared with the control (21%).

Planned pairwise contrasts focusing on the Momentum intervention revealed that at T3, participants in the Momentum condition were significantly more likely to report participation than those in the Control condition (*OR* = 1.96, *p* = .032) and the Moral Harm condition (*OR* = 1.97, *p* = .041). No significant differences emerged at earlier time points.

In sum, removing the covariates did not substantively alter the pattern of results. The Momentum intervention continued to attenuate the decline in participation observed in the Control condition, strengthening confidence in the robustness of the main findings

**generalized linear mixed-effects model**

An additional analysis was conducted using a generalized linear mixed-effects model (GLMM) with a logit link to account for the longitudinal structure of the data and handle missing observations under the Missing at Random assumption. The model included a random intercept for participants and fixed effects for time (three measurement points), experimental condition, the Time × Condition interaction, and covariates for age, political ideology, and exposure.

Results indicated substantial between-subject variability (*SD* = 2.35) and an overall decline in the likelihood of participation over time (*b*ₜ₂ = −.75, *SE* = .26, *p* = .004; *b*ₜ₃ = −1.05, *SE* = .33, *p* = .002). Importantly, a significant Time × Momentum interaction was found at the final measurement point (*b* = 1.05, *SE* = .47, *p* = .026), suggesting that participants in the Momentum condition were more likely to engage in protest activities at follow-up than those in the control group.

Estimated marginal probabilities confirmed this pattern: while groups did not differ at baseline (T1; *p*s > .85), the Momentum group showed higher participation rates after two and a half months (T3; *OR* = 3.01 vs. Control, *p* = .046; *OR* = 3.48 vs. Moral Harm, *p* = .026). A sensitivity analysis using a generalized estimating equation (GEE) with an exchangeable correlation structure yielded consistent results (*b* = .62, *SE* = .31, *p* = .043).

We also tested a model with a random slope for time, but it produced a singular fit (random slope variance ≈ 0, *χ²* = .04, *p* = .84 compared to the random-intercept model). Therefore, the simpler random-intercept model was retained. Results and conclusions remained unchanged, with a significant Time × Momentum interaction at T3.

Overall, this longitudinal mixed-model analysis supports the findings reported in the main manuscript: the Momentum-based intervention did not produce immediate effects but significantly enhanced sustained engagement over time, even when accounting for within-subject correlations and incomplete longitudinal data.

**Attrition Analysis**

An attrition analysis was conducted to assess potential dropout bias across experimental conditions and determine whether dropout rate differences could influence the study’s outcomes. Dropout was defined as participants who underwent the interventions at T2 but did not participate at subsequent time points, T3. The decline in response rate at T3 is attributed to the small sample size taken from the original group at T1. Participation invitations were sent to all participants, but data collection ended once quotas for political ideology and gender were met. Consequently, some participants who did not complete the survey may have done so if given the opportunity. Therefore, we checked for dropout biases.

***Dropout Rates by Experimental Condition***

The dropout rates for each experimental condition are presented in Table S1.

The dropout rates ranged from 40.19% in the Control condition to 51.01% in the Momentum condition. A chi-square test indicated that the dropout rate in the Momentum condition was significantly higher than in the Control condition, *χ²*(1, *N* = 609) = 4.82, *p* = .028. This finding raises concerns about potential attrition bias affecting the study’s results.

***Predictors of Dropout***

Logistic regression analyses were conducted to investigate potential predictors of dropout. The first model included experimental condition, age, gender, political ideology, number of interventions’ exposures, past participation as measured at T1, income, and religiosity as predictors. The results are summarized in Table S2.

Participants in the Momentum condition had significantly higher odds of dropping out than the Control condition (*B* = .453, *SE* =.170, *z* = 2.658, *p* = .008). Age was a significant negative predictor of dropout, indicating that older participants were less likely to drop out (*B* = -.0097, *SE* = .0041, *z* = -2.330, *p* = .020). Political ideology was a significant positive predictor, suggesting that participants with a slight tendency towards left-leaning political views were likelier to drop out (*B* = .178, *SE* = .0569, *z* = 3.126, *p* = .002). Exposure was also a significant negative predictor, with higher exposure associated with lower dropout rates (*B* = -.433, *SE* = .0857, *z* = -5.057, *p* < .001).

An interaction model was also tested to examine whether the effect of participant characteristics on dropout varied by condition. Notably, the interaction between the Moral Harm condition and political ideology was significant (*B* = -.368, *SE* = .1641, *z* = -2.241, *p* = .025), suggesting that political ideology influenced dropout differently in the Moral Harm condition compared to the Control condition.

***Comparison of Dropouts and Non-Dropouts***

Independent samples t-tests and chi-square tests were conducted within each condition to determine whether dropouts differed from completers on key variables. P-values were adjusted using the Bonferroni correction to account for multiple comparisons.

**Continuous Variables**

The results of the *t*-tests for continuous variables are presented in Table S3.

After adjusting for multiple comparisons, significant differences remained in the Control condition for political ideology (*adjusted p* = .0076) and in the Democratic Identity condition for exposures (*adjusted p* = .00075). Dropouts in the Control condition had a slight tendency towards right-leaning political views than non-dropouts, and dropouts in the Democratic Identity condition had higher numbers of exposures.

***Categorical Variables***

Chi-square tests were conducted for categorical variables, and the results are presented in Table S4.

**Analysis Using Only Participants Who Completed All Time Points**

To address the concern that the higher dropout rate in the Momentum condition might influence the observed treatment effects, an analysis was conducted using only participants who underwent the interventions and completed T3 (completers).

A logistic regression model fitted with protest participation as the dependent variable, including condition, time, and their interaction, controlling for exposures, age, and political ideology, as in the main analysis. Estimated marginal means were calculated, and pairwise comparisons were made using the Bonferroni correction to account for multiple comparisons. The results are presented in Table S5.

Pairwise comparisons revealed that at T3, participants in the Momentum condition had a significantly higher probability of protest participation compared to those in the Control condition (*OR* = 2.13, *SE* = .610, *z* = 2.627, *p* = .0258, Bonferroni-adjusted). Similarly, the Momentum condition showed a higher probability than the Moral Harm condition (*OR* = 2.49, *SE* = .756, *z* = 3.017, *p* = .0077).

**Results Discussion**

The attrition analysis revealed that the Momentum condition had a higher dropout rate, which could potentially bias the study’s findings. However, logistic regression analyses indicated that while the Momentum condition predicted higher dropout, other factors such as age, political ideology, and exposure also played significant roles.

Importantly, when comparing dropouts to non-dropouts within conditions, significant differences were limited and, after adjusting for multiple comparisons, primarily involved exposure and political ideology in specific conditions. This suggests that dropout did not systematically differ on key variables across conditions.

The analysis restricted to participants who completed T3 supported the results found in the main analysis. Even when accounting for potential attrition bias by excluding dropouts, the Momentum condition continued to show a significant increase in protest participation compared to other conditions. Moreover, all variables that were found significant in the dropout analysis, in any of the tests, were controlled in the main analysis.

Table S1. Dropout Rates by Experimental Condition.

| **Condition** | **Total Participants** | **Dropouts** | **Dropout Rate (%)** |
| --- | --- | --- | --- |
| Control | 311 | 125 | 40.19 |
| Democratic Identity | 303 | 128 | 42.38 |
| Momentum | 298 | 152 | 51.01 |
| Moral Harm | 306 | 142 | 46.41 |

Table S2. Logistic Regression Predicting Dropout Status.

| **Predictor** | **B** | **SE** | ***z*** | ***p*** |
| --- | --- | --- | --- | --- |
| Intercept | .471 | .433 | 1.086 | .278 |
| Democratic Identity | .092 | .171 | .538 | .591 |
| Momentum | .453 | .170 | 2.658 | **.008** |
| Moral Harm | .332 | .172 | 1.936 | .053 |
| Age | -<.001 | .004 | -2.330 | *.020* |
| Gender (Female) | -.037 | .123 | -.304 | .761 |
| Political Ideology | .178 | .057 | 3.126 | **.002** |
| Exposures | -.433 | .086 | -5.057 | ***<.001*** |
| Past Participation at T1 (Yes) | -.072 | .140 | -.512 | .608 |
| Income | -<.001 | .051 | -.005 | .996 |
| Religiosity | .010 | .064 | .158 | .874 |

*Note*. *p* < .05; **p** < .01; ***p*** < .001.

**Table S3.** Comparison of Continuous Variables Between Dropouts and Non-Dropouts by Condition.

| **Condition** | **Variable** | ***t* (df)** | ***p*** | **Mean Dropout** | **Mean No Dropout** | **Adjusted *p*** |
| --- | --- | --- | --- | --- | --- | --- |
| Control | Age | 1.48 (292) | .140 | 43.6 | 40.9 | .560 |
| Democratic Identity | Age | 2.30 (286) | *.022* | 46.3 | 42.2 | .088 |
| Momentum | Age | -0.31 (286) | .760 | 43.6 | 44.1 | 1.000 |
| Moral Harm | Age | .68 (281) | .495 | 45.1 | 43.9 | 1.000 |
| Control | Political Ideology | -3.14(292) | **.002** | 3.92 | 4.38 | *.0076* |
| Democratic Identity | Political Ideology | -1.02(286) | .308 | 4.08 | 4.23 | 1.000 |
| Momentum | Political Ideology | -1.00(286) | .316 | 3.99 | 4.14 | 1.000 |
| Moral Harm | Political Ideology | -.13(281) | .895 | 4.08 | 4.10 | 1.000 |
| Control | Exposures | 1.52 (292) | .130 | 2.51 | 2.38 | .522 |
| Democratic Identity | Exposures | 3.79 (286) | ***<.001*** | 2.59 | 2.27 | ***<.001*** |
| Momentum | Exposures | 1.67 (286) | .095 | 2.51 | 2.38 | .382 |
| Moral Harm | Exposures | 2.78 (281) | **.006** | 2.55 | 2.32 | *.023* |

*Note*. Adjusted *p*-values computed using Bonferroni correction. *p* < .05; **p** < .01; ***p*** < .001.

**Table S4.** Chi-Square Tests for Categorical Variables by Condition.

| **Condition** | **Variable** | **χ² (df)** | ***p*** | **Adjusted *p*** |
| --- | --- | --- | --- | --- |
| Control | Gender | .024(1) | .877 | 1.000 |
| Democratic Identity | Gender | .878(1) | .349 | 1.000 |
| Momentum | Gender | .019(1) | .889 | 1.000 |
| Moral Harm | Gender | 1.034(1) | .309 | 1.000 |
| Control | Past Participation | 1.897(1) | .168 | .674 |
| Democratic Identity | Past Participation | .000(1) | 1.000 | 1.000 |
| Momentum | Past Participation | .000(1) | 1.000 | 1.000 |
| Moral Harm | Past Participation | .089(1) | .766 | 1.000 |

No significant differences were found for gender or past participation after adjusting for multiple comparisons.

**Table S5.**  Estimated Probabilities of Protest Participation by Condition and Time Among Completers.

| **Time** | **Condition** | **Probability** | ***SE*** | **95% *CI*** |
| --- | --- | --- | --- | --- |
| 1 | Control | .436 | .043 | [.355, .520] |
|  | Democratic Identity | .481 | .044 | [.396, .567] |
|  | Momentum | .508 | .048 | [.415, .600] |
|  | Moral Harm | .506 | .047 | [.415, .597] |
| 2 | Control | .210 | .034 | [.152, .284] |
|  | Democratic Identity | .231 | .035 | [.169, .307] |
|  | Momentum | .307 | .044 | [.227, .399] |
|  | Moral Harm | .143 | .029 | [.094, .211] |
| 3 | Control | .175 | .023 | [.124, .241] |
|  | Democratic Identity | .233 | .035 | [.172, .308] |
|  | Momentum | .310 | .043 | [.233, .400] |
|  | Moral Harm | .153 | .0297 | [.103, .220] |

**Table S6.**

Content validity (I-CVI, S-CVI/Ave) and inter-expert agreement (Kendall’s W) for the three manipulations (N = 19 experts)

| **Manipulation** | **Item** | **I-CVI** | **S-CVI/Ave** | **Kendall’s W** | ***p*** |
| --- | --- | --- | --- | --- | --- |
| **Identity** | “Belonging to a democracy-supporting group” | .95 | .88 | .15 | .06 |
|  | “The protest is a way to defend democratic identity” | .74 |  |  |  |
|  | “Participating in the protest is part of being democratic” | .95 |  |  |  |
| **Moral harm** | “The reform harms basic moral values” | .90 | .83 | .16 | .05 |
|  | “Participating in the protest is a moral duty” | .68 |  |  |  |
|  | “Participation defends moral principles” | .90 |  |  |  |
| **Momentum** | “The protest is gaining momentum” | .95 | .83 | .44 | < .001 |
|  | “The protest is getting closer to its goals” | .58 |  |  |  |
|  | “The protest is growing and reaching new audiences” | .95 |  |  |  |

Note. I-CVI values represent the proportion of experts who rated each item as relevant (4–6 on a 1–6 scale). S-CVI/Ave is the average I-CVI across the three items within each manipulation, with values ≥ .80 typically interpreted as indicating good content validity. Kendall’s W indexes agreement among experts regarding the relative ranking of items within each manipulation. Lower W values for the democratic identity and moral-harm items (≈ .15) reflect that experts tended to rate all three items within each construct as similarly relevant, whereas the higher W for the momentum items (*W* = .44, *p* < .001) indicates stronger agreement that some momentum items were more representative than others.

**Table S7.**

The demographic characteristics of the full sample, as well as separately for each experimental condition.

| Condition | N | Age Mean | Age SD | Ideology Mean | Ideology SD | % Male | % Other/Unknown |  |
| --- | --- | --- | --- | --- | --- | --- | --- | --- |
| control | 311 | 42.71 | 15.71 | 4.12 | 1.27 | 44.05 | .00 |  |
| Democrat identity | 303 | 44.03 | 15.01 | 4.14 | 1.32 | 53.14 | .00 |  |
| momentum | 298 | 43.62 | 15.10 | 4.07 | 1.29 | 47.65 | .34 |  |
| Moral harm | 306 | 44.14 | 14.48 | 4.09 | 1.30 | 53.27 | .00 |  |
| Total | 1,218 | 43.62 | 15.07 | 4.11 | 1.30 | 49.51 | .08 |  |
| Notes: Political ideology measured 1 to 7 (1 = Extreme right, 7 = Extreme left). Percent columns are within-condition percentages. | | | | | | | | |

**Table S8.**

An ANOVA treating exposure as continuous confirmed that mean exposure did not differ significantly across conditions, *F*(3, 1214) = .15, *p* = .93.

A Pearson chi-squared treating exposure as categorical showed significant differences *χ²*(6, *N* = 1218) = 18.031, *p* = .006

| Condition | Exposure = 1 | Exposure = 2 | Exposure = 3 | Total |
| --- | --- | --- | --- | --- |
| control | 61 | 63 | 187 | 311 |
| democrat_identity | 44 | 94 | 165 | 303 |
| momentum | 39 | 100 | 159 | 298 |
| moral_harm | 48 | 97 | 161 | 306 |
| Total | 192 | 354 | 672 | 1,218 |
|  | | | | |

**Table S9**Balance checks across experimental conditions among T2d respondents

| Variable | Control (n=286) | Democratic identity (n=280) | Momentum (n=280) | | Moral harm (n=275) | *F* (df1, df2) | *p* |
| --- | --- | --- | --- | --- | --- | --- | --- |
| Age | 42.42 (15.71) | 44.40 (15.09) | 43.83 (14.92) | 44.46 (14.56) | | 1.13 (3, 1117) | .338 |
| Political ideology | 4.13 (1.29) | 4.17  (1.32) | 4.08 (1.28) | 4.09 (1.31) | | .31 (3, 1117) | .820 |
| Exposure count | 2.47 (.76) | 2.46  (.69) | 2.46 (.68) | 2.44  (.69) | | .07 (3, 1117) | .978 |
| Income | 2.92 (1.28) | 3.00  (1.21) | 3.07 (1.22) | 2.99 (1.23) | | .69 (3, 1117) | .559 |

*Note.* Cells show M (SD). One-way ANOVAs test differences across conditions; df2 reflects residual degrees of freedom for each model (*N* = 1121).

**Table S10**Balance checks across experimental conditions among T3 respondents

| Variable | Control (n=169) | Democratic identity (n=161) | Momentum (n=136) | Moral harm (n=141) | *F* (df1, df2) | *p* |
| --- | --- | --- | --- | --- | --- | --- |
| Age | 43.60 (15.99) | 45.96 (14.67) | 43.60 (15.32) | 45.11 (14.88) | .92 (3, 603) | .429 |
| Political ideology | 3.92 (1.28) | 4.07  (1.31) | 3.99  (1.31) | 4.08  (1.35) | .55 (3, 603) | .646 |
| Exposure count | 2.51 (.74) | 2.59  (.63) | 2.51  (.63) | 2.55  (.65) | .48 (3, 603) | .694 |
| Income | 2.88 (1.29) | 2.99  (1.28) | 3.06  (1.29) | 3.03  (1.24) | .62 (3, 603) | .602 |

*Note.* Cells show M (SD). (*N* = 607). One-way ANOVAs test differences across conditions (*N* = 607).

**Table S11**Logistic regression predicting past protest participation at baseline from condition

| Predictor | *B* | *SE* | *z* | *p* | *OR* | 95% *CI* for *OR* |
| --- | --- | --- | --- | --- | --- | --- |
| Intercept (Control) | -.674 | .120 | -5.620 | < .001 | .51 | [.40, .64] |
| Democratic identity vs Control | .025 | .170 | .147 | .883 | 1.03 | [.73, 1.43] |
| Momentum vs Control | .006 | .171 | .034 | .973 | 1.01 | [.72, 1.41] |
| Moral harm vs Control | -.139 | .172 | -.808 | .419 | .87 | [.62, 1.22] |

*Note.* Outcome is any past protest participation at baseline. Control is the reference condition. Likelihood ratio test for the full model: *χ²*(3) = 1.140, *p* = .767.


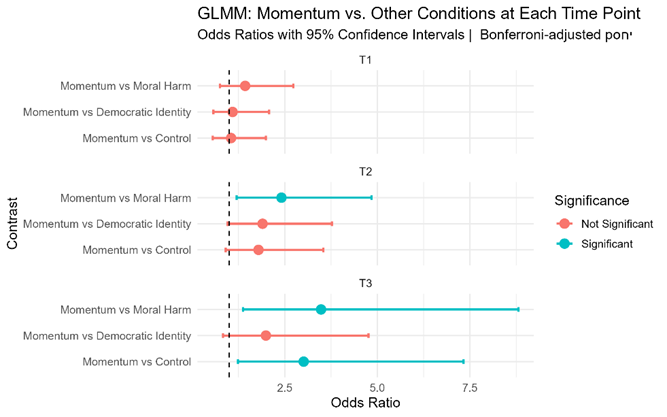


**Figure S1.** Generalized Linear Mixed Model (GLMM) results comparing the Momentum condition to other intervention conditions across time points (T1–T3). Displayed are odds ratios (OR) with 95% confidence intervals; p-values are Bonferroni-adjusted.”

Appendix 1.

**The script of the momentum video:**

Not just lawyers

Economists

High-Techists

Doctors

Pilots

Academics

Military reserves

Women

Young people

Secular

Religious

Leftists

Rightists

Not only in Tel Aviv

In Jerusalem

In Be’er Sheva

In Ashdod

In Haifa

On roads

At intersections

In squares

On bridges

In Ben Gurion Airport

Not only you.

not only us.

All of us!

Week by week we grow, and the protest is getting stronger

Hundreds of thousands more take to the streets, in every city, everywhere

And give the protest the momentum it needs

TO STOP THE REFORM

Join the democratic action that sweeps the streets

**The script of the democratic identity video:**

I am secular

I am religious

I am a woman

I am a man

I am Sephardic Jew

I am Ashkenazic Jew

I am leftist

I am rightist

I am from Be’er Sheva

I am from Be’er Tobiah

I am democratic

We are joining the protest

EQUAL RIGHTS

FREEDOM OF SPEECH

PROTECTING MINORITY GROUPS

STATE INSTITUTIONS

AND THE PURITY OF MORALS

We are democrats

Without democracy there is no us

I am democratic

We are joining the protest

**The script of the moral harm video:**

Why join the protest?

Because a reform that harms democracy

VIOLATES EQUAL RIGHTS

VIOLATES FREEDOM OF SPEECH

HARMS MINORITY GROUPS

AND HARMS THOSE WHOM ONLY A TRUE DEMOCRACY WILL PROTECT FROM A PREDATORY MAJORITY

Therefore - we are not allowed to give up!

Why join the protest?

Because a reform that harms democracy

HARMS THE JEWISH AND DEMOCRATIC STATE OF ISRAEL

HARMS STATE INSTITUTIONS

AND HARMS THE PURITY OF MORALS

Therefore - we are not allowed to give up!

Why join the protest?

Because there is no such thing as half democracy

There is no such thing as a democracy with “compromises”

Therefore - join the protest!

Appendix 2.

**Momentum poster:**

Not only

Lawyers, Economists, High-Techists, Doctors,

Pilots, Military Reservists, Academics, Women,

Youth, Elderly, Secular, Religious, Leftist, Rightist

Hundreds of thousands take to the streets

And give the protest the momentum it needs to stop the reform

Join the democratic action that sweeps the streets!

**Democratic identity poster:**

Equal Rights, Freedom of Speech, Protecting Minority Groups, State Institutions and the Purity of Morals IS US

I am democratic

Without democracy there is no us

We are joining the protest!

**Moral harm poster:**

Why join the protest?

Because the reform harms the Jewish and democratic state of Israel, violates equal rights, violates the freedom of speech, harms minority groups, harms state institutions, and harms the purity of morals

THERE IS NO SUCH THING AS A DEMOCRACY WITH “COMPROMISES”

Those who are loyal to safeguarding the rights of all citizens

Those who are loyal to a Jewish and democratic state

join the protest!

1. During the period between T1 and T2, the same group of subjects was given another questionnaire. This questionnaire included an experiment that involved only written messages as part of a different study. No significant differences were observed between the groups or any noticeable effects. The complete questionnaire can be found in the study's OSF project reports https://osf.io/xmkpf. [↑](#footnote-ref-1)
